# Seasonality and environmental determinants of exhaled nitric oxide in individuals with and without chronic respiratory diseases

**DOI:** 10.1097/EE9.0000000000000501

**Published:** 2026-06-30

**Authors:** Lorena Torroni, Francesca Locatelli, Pierpaolo Marchetti, Sandra Baldacci, Claudio Gariazzo, Sara Maio, Camillo Silibello, Gianluca Spiteri, Massimo Stafoggia, Giovanni Viegi, Giuseppe Verlato, Alessandro Marcon

**Affiliations:** a Unit of Epidemiology and Medical Statistics, Department of Diagnostics and Public Health, University of Verona, Verona, Italy; b Institute of Clinical Physiology, National Research Council (CNR), Pisa, Italy; c Occupational and Environmental Medicine, Epidemiology and Hygiene Department, Italian Workers’ Compensation Authority (INAIL), Roma, Italy; d ARIANET s.r.l., Milano, Italy; e Occupational Medicine Unit, University Hospital of Verona, Verona, Italy; f Department of Epidemiology, Lazio region Health Service, ASL ROMA1, Roma, Italy; g Institute of Translational Pharmacology, National Research Council (CNR), Palermo, Italy

**Keywords:** Air pollution, Air temperature, Airway inflammation, Environmental epidemiology, Fractional exhaled nitric oxide (FeNO), Asthma, Rhinitis

## Abstract

**Background and Objective:**

Fractional exhaled nitric oxide (FeNO) is a biomarker of type-2 lung inflammation. Standardized measurement is essential for accurate diagnosis and monitoring. We assess seasonality and environmental determinants of FeNO in individuals with and without chronic respiratory diseases in the general population.

**Methods:**

This is a cross-sectional study on 412 individuals with chronic respiratory diseases (asthma, chronic bronchitis/chronic obstructive pulmonary disease, rhinitis) and 605 individuals without these conditions. Participants, aged 20–65 years, were recruited in the Gene-Environment Interactions in Respiratory Diseases study in Verona, Italy (2008–2014). Geocoded residential addresses were linked to daily PM_10_ and air temperature at the time of the clinical examination using previously developed spatiotemporal models. Associations with log-FeNO were analyzed using adjusted linear regression, accounting for seasonality and disease status.

**Results:**

FeNO levels were higher in subjects with respiratory diseases during the warm season, even after adjusting for pollen exposures; a milder seasonal pattern was observed in subjects without chronic respiratory diseases (*P* for interaction = 0.001). A 10 μg/m^3^ increase in mean PM_10_ concentration at lag 0–1 (day of FeNO measurement and day before) was associated with a 3% higher FeNO concentration (Ratio of Geometric Means, RGM: 1.03, 95% confidence interval: 1.00, 1.06) after adjusting for seasonality.

**Conclusion:**

FeNO is a sensitive biomarker of environmental exposures. Overlooking seasonality and environmental factors might impact clinical decision-making in chronic respiratory diseases.

**What this study adds**

This study shows that FeNO, a biomarker of type-2 airway inflammation, is influenced by both seasonality and short-term environmental exposures in a population-based adult cohort. FeNO levels were higher during the warm season, even among individuals without chronic respiratory diseases, after adjusting for pollen exposure, and increased with concurrent PM_10_ exposure. By jointly examining seasonal patterns alongside short-term environmental exposures, our findings highlight the importance of accounting for environmental variability when interpreting FeNO measurements in epidemiological research and clinical practice. These results are directly relevant to environmental epidemiology, as they support the use of FeNO as a sensitive biomarker of environmentally driven respiratory changes.

## Introduction

Particulate matter (PM_10_ and PM_2.5_), nitrogen dioxide (NO_2_), and ozone (O_3_) are among the most dangerous air pollutants. In 2022, the proportion of the European urban population exposed to levels of these pollutants exceeding the WHO 2021 guidelines ranged from 83% (PM_10_) to 96% (PM_2.5_).^1^

Air pollutants can trigger proinflammatory activity and oxidative stress, resulting in long-term outcomes such as asthma and chronic obstructive pulmonary disease (COPD),^2,3^ as well as acute episodes that require additional therapy or necessitate hospitalization. The mortality linked to chronic air pollution across the 27 European Union countries is substantial.^4^ In Italy, PM_2.5_ exposure was responsible for more than 72,000 annual deaths during 2016–2019.^5^ Air pollution also increases morbidity and entails significant healthcare costs. In Italy, PM_2.5_ exposure above the 2021 WHO guideline was associated with 23,544 years lived with disability due to COPD in 2023.^6^ In Italy, short-term exposure to PM_10_ and PM_2.5_ has been associated with increases in respiratory admissions of 1.20% and 1.22%, respectively, per 10 µg/m^3^ increments in exposure at lag 0–5.^7^

Temperature fluctuations are also linked to adverse respiratory effects in susceptible individuals, including increased episodes of bronchospasm, hospital admissions, and mortality due to worsening respiratory conditions.^7–11^ In healthy individuals, exposure to extreme temperatures can cause distress and respiratory infections. Prolonged exposure may also increase the risk of developing chronic diseases.^12^

The fractional concentration of exhaled nitric oxide (FeNO) is a noninvasive biomarker of type-2 airway inflammation, reflecting nitric oxide (NO) production by the respiratory epithelium through the activation of inducible NO synthase in response to triggers.^13^ FeNO is commonly measured to detect and monitor lung inflammation. International guidelines recommend performing FeNO measurements under controlled environmental conditions.^14^ In recent years, growing but conflicting evidence has suggested that short-term exposure to air pollution may influence FeNO levels.^15,16^ The effects of seasonal temperature variations on respiratory health have been widely studied, but fewer studies have examined airway inflammation and day-to-day temperature variability.

However, several gaps remain. Most studies have focused on children or elderly populations, often in highly polluted settings, and have rarely compared individuals with and without chronic respiratory diseases. In addition, although temporal trends are often controlled for in the analysis, seasonal patterns and multiple short-term environmental exposures have not always been examined jointly.

Addressing these limitations may improve the interpretation of FeNO as a biomarker of airway inflammation in both clinical and epidemiological contexts.

## Aim

This study aims to evaluate whether FeNO concentrations exhibit seasonal patterns among adult Italian subjects with and without chronic respiratory diseases, recruited from the general population. Additionally, we assess how variations in residential exposure to air pollution and air temperature affect FeNO concentrations.

## Methods

### Study design and sampling

**Figure 1.**
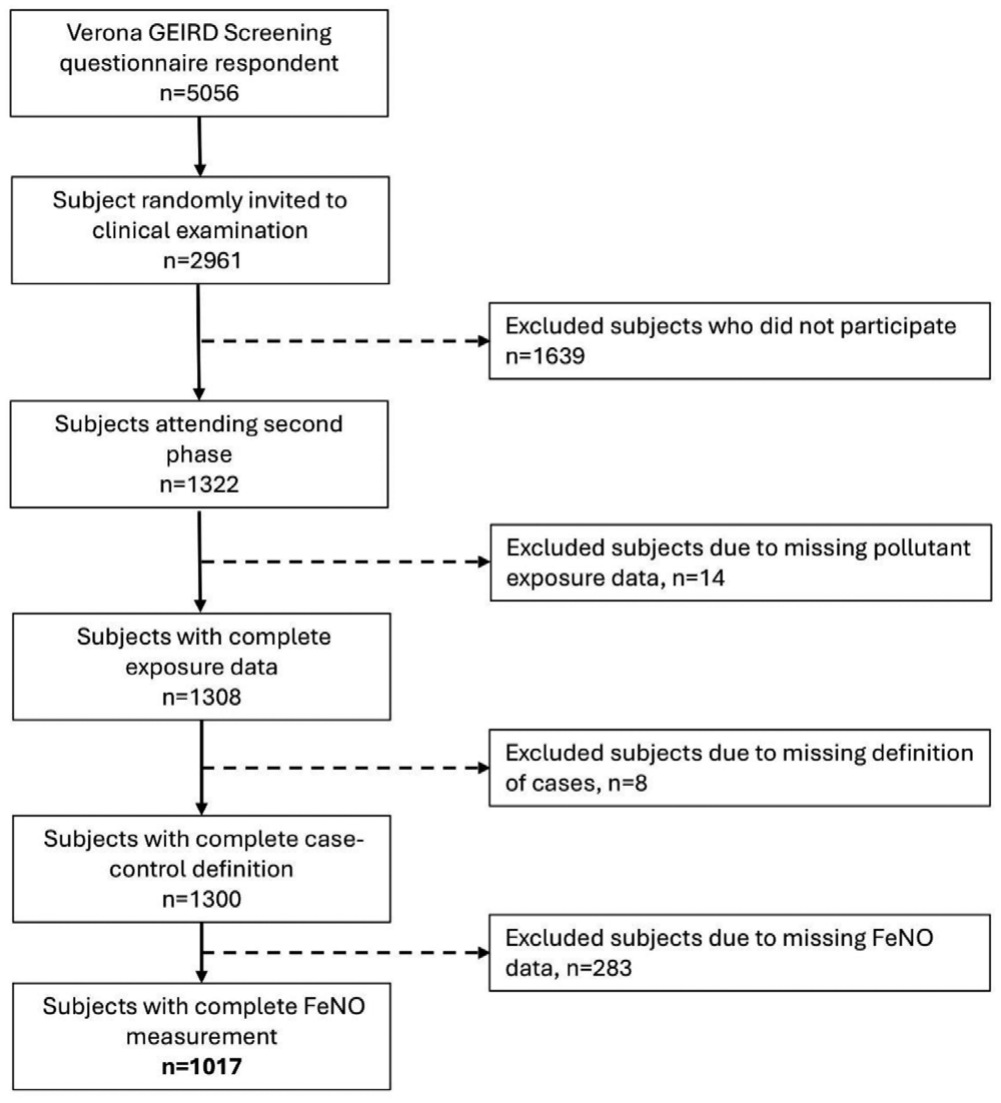
Flow chart of study participants.

This study used data from individuals living in the city of Verona who participated in the Genes-Environment Interactions in Respiratory Diseases (GEIRD) study.^17^ Participants were identified through a two-phase screening process from preexisting random cohorts or a new random sampling of the general Italian population (**Figure 1**).^18^

The first phase of GEIRD was a screening questionnaire survey on self-reported respiratory symptoms, administered to random samples of individuals aged 20–64 between 2007 and 2010.

A multicase-control study was implemented in the second phase (2008–2016). All participants reporting symptoms suggestive of asthma, chronic bronchitis, or COPD in the screening questionnaire, as well as a 30% random sample of subjects with rhinitis (“probable cases”), were invited to undergo a clinical examination. A 40% random sample of individuals who did not report respiratory symptoms on the screening questionnaire (“probable controls”) was also selected from the same base population. No matching criteria were applied.

At the clinical examination, all participants underwent a structured interview adapted from the European Community Respiratory Health Survey questionnaire.^19^ Height and weight were measured. Skin-prick tests and FeNO measurements were conducted as described below.

### Definitions

Allergic sensitization was defined as a positive skin-prick test to inhaled allergens (**Supplementary Methods**; https://links.lww.com/EE/A438).

Consistent with a previous publication,^18^ subjects were classified using the following hierarchical definition based on their responses to the clinical questionnaires:

- Asthma (with/without rhinitis or chronic bronchitis/COPD): subjects reporting a lifetime diagnosis of asthma;
- Chronic bronchitis/COPD (with/without rhinitis, but without asthma): subjects who had a cough for >3 months/year for ≥2 years and/or phlegm for >3 months/year for ≥2 years and/or a self-reported diagnosis of chronic bronchitis, COPD, or emphysema;
- Rhinitis (with neither asthma nor chronic bronchitis/COPD): subjects who reported lifetime nasal allergies, including “hay fever” at the time of the survey or a problem with sneezing or a runny or blocked nose without cold/flu in the last 12 months;
- None of these diseases: subjects who did not meet the above disease definitions (reference group).^18^

### Fractional exhaled nitric oxide

FeNO measurements were performed at a flow rate of 50 mL/s, expressed in parts per billion (ppb), before spirometry, in accordance with international guidelines, using a chemiluminescence analyzer (CLD88, Eco Medics, Switzerland). Subjects were instructed to take a deep breath, hold it briefly, then slowly exhale into a mouthpiece fitted with a 0.2-μm bacterial filter. NO was directly sampled into the analyzer at 250 mL/min through a Teflon side-arm tube connected to the sampling port (**Supplementary Methods**; https://links.lww.com/EE/A438).

### Exposures estimated at residential addresses

The residential street addresses of all the participants were geocoded.

Time series of daily concentrations of the primary air pollutants (PM_10_, PM_2.5_, NO_2_, and O_3_) and air temperature were associated with the geocoded individual addresses. These time series were obtained using validated models previously developed in the BEEP (Big data in Environmental and Occupational Epidemiology) and BIGEPI (use of big data to assess the acute and chronic health effects of air pollution in the Italian population; https://bigepi.it/) projects at a 1-km resolution.^20^ In brief, daily PM_10_ (years 2006–2015) and PM_2.5_ (years 2013–2015) concentrations were estimated using spatiotemporal models based on machine-learning random forest algorithms.^21,22^ Daily NO_2_ and O_3_ concentrations (years 2013–2015) were derived using an integrated approach that coupled a chemical transport model with machine-learning techniques.^18,23^ Daily mean air temperature (2006–2015) was estimated at a 1-km resolution using a mixed-effects regression model, in which measurements from meteorological stations were calibrated against land surface temperature and other spatial (e.g., vegetation index, land use, and geoclimatic characteristics) and temporal predictors (e.g., seasonality and relative humidity).^24,25^ Cross-validated R^2^ were 0.75 (PM_10_), 0.81 (PM_2.5_), 0.60 (NO_2_), 0.80 (O_3_), and 0.95 (temperature), demonstrating good predictive performance of the exposure models.^21–23^

Finally, we averaged daily pollutant concentrations over different windows depending on the temporal scale of the analysis. We investigated the short-term effects of PM_10_ and air temperature, using latency intervals of two days (lag 0–1), four days (lag 0–3), and seven days (lag 0–6) before the date of FeNO measurement. These lag intervals were chosen a priori based on biological plausibility to assess immediate, delayed, and prolonged effects.^26^ We did not evaluate the short-term effects of PM_2.5_, NO_2_, and O_3_ because their daily time series did not cover the period of FeNO measurements.

We investigated long-term associations using exposure indicators obtained by averaging NO_2_, PM_2.5_, PM_10,_ summer O_3_ concentrations, and air temperature over the period 2013–2015, a common period for all four pollutants.

### Exposures estimated at the area level

Daily air concentrations of seven pollen species (Poaceae, Urticaceae, Oleaceae, Cupressaceae, Coryloideae, *Betula,* and *Ambrosia*) were obtained from a monitoring station located within the city for the years corresponding to the clinical examinations.^27^ Similarly, daily data on relative humidity and barometric pressure were obtained from the closest meteorological monitoring stations (Villafranca di Verona and Bardolino Calmasino, respectively). These measurements were not available at the individual residential level; therefore, the same values were assigned to all participants based on the date of the clinical examination. Specifically, pollen concentrations were averaged over the week preceding the clinical examination, as in a previous publication.^27^ A participant was considered exposed to a specific pollen if the mean concentration exceeded the threshold set by the Italian Aerobiological Monitoring Network of the Italian Association of Aerobiology.^28^ For relative humidity and barometric pressure, the averaging periods were aligned with the main analysis (lag 0–1, lag 0–3, and lag 0–6).

### Statistical analysis

Descriptive statistics were reported for exposure variables using mean (±standard deviation [SD]), median, range (min–max), and interquartile range (I–III quartile). Correlations between exposure variables were assessed using Spearman’s correlation coefficient.

FeNO values were log-transformed to fulfill the assumption of normality. We investigated the intra-annual variation (seasonality) in log-FeNO using multiple linear regression models, incorporating both a linear (day) and a quadratic (day^2^) term for the day of the year when FeNO was measured to capture potential nonlinear seasonal patterns. The day variable, ranging from 1 to 365, was centered at its mean before computing the squared term to mitigate structural multicollinearity between the linear and quadratic terms. This approach improves the stability and interpretability of estimated regression coefficients.

Models were adjusted for sex, age, body mass index (BMI), smoking habits, and allergic sensitization (**Table 1**). Missing data were deleted listwise since their occurrence was limited (**Table 1**). We included disease status as a design variable to account for the sampling structure. Due to the insufficient number of participants in separate disease groups to estimate disease-specific seasonal patterns, the analysis included a binary disease status indicator (i.e., individuals with and without asthma, chronic bronchitis/COPD, and rhinitis). Interaction terms for day × disease status and day^2^ × disease status were included to allow seasonal patterns to differ between the two groups. This approach was used because stratification by disease status was not feasible due to the small sample size. Seasonality was illustrated graphically using marginal estimates from this model.

**Table 1.** Characteristics of the participants by disease status

	Subjects without chronic respiratory diseases n = 605 (%)	Subjects with chronic respiratory diseases^a^ n = 412 (%)	*P*-value
Age			
20–39 years	160 (26.4)	140 (34.0)	<0.001
40–49 years	225 (37.2)	165 (40.0)	
50–65 years	220 (36.4)	107 (26.0)	
Male sex	298 (49.3)	215 (52.2)	0.359
Full-time education			
0–8 years	143 (23.6)	91 (22.1)	0.827
9–13 years	162 (26.8)	115 (27.9)	
≥14 years	300 (49.6)	206 (50.0)	
Smoking habits			
Nonsmoker	293 (48.6)	205 (49.8)	0.857
Former smoker	178 (29.5)	115 (27.9)	
Current smoker	132 (21.9)	92 (22.3)	
Missing data	2	-	
Pack years smoked			
0	293 (49.2)	205 (50.4)	0.582
<15	182 (30.5)	130 (31.9)	
≥15	121 (20.3)	72 (17.7)	
Missing data	9	5	
BMI			
Underweight	9 (1.5)	7 (1.7)	0.270
Normal weight	313 (51.9)	237 (57.5)	
Overweight	205 (34.0)	128 (31.1)	
Obese	76 (12.6)	40 (9.7)	
Missing data	2	-	
Allergic sensitization			
Present	198 (34.2)	314 (78.7)	<0.001
Missing data	6	13	

a The 412 subjects with chronic respiratory diseases included 152 with asthma (with/without rhinitis or chronic bronchitis/COPD), 63 with bronchitis/COPD (with/without rhinitis, but without asthma), and 197 with rhinitis (alone).

To assess the impact of pollen exposures on FeNO seasonality, an additional analysis was conducted, including a composite indicator of pollen exposure during the week before FeNO measurement and individual sensitization to the relevant pollen species (**Supplementary Figure 1**; https://links.lww.com/EE/A438).^29^

The short-term associations between exposure variables and log-FeNO were first analyzed considering PM_10_ concentrations and temperature as predictors in separate models, adjusting for sex, age, BMI, smoking habits, allergic sensitization, day, day^2^, and disease status. The hierarchical disease indicator was included in this analysis to account for differences between groups and for the stratified sampling scheme. Then, mutually adjusted associations were described, including PM_10_ concentrations and temperature in the same model. Association estimates were expressed as ratios of geometric means (RGM) with 95% confidence intervals (CIs) per 10 μg/m^3^ increment in PM_10_ and 10 °C increment in air temperature. RGM and CIs were obtained by exponentiating the regression coefficients and their confidence limits, thus representing the multiplicative change in the geometric mean of FeNO associated with the specified exposure increments.

We also conducted sensitivity analyses: (1) to better capture the different FeNO seasonality between subjects with and without chronic respiratory diseases, we included the interactions of day and day^2^ with disease status, using the binary disease indicator due to sparse data; (2) to test whether differences in recruiting participants over time could bias the results, year was included as an additional categorical adjustment variable; (3) to evaluate the impact of meteorology, we further adjusted for relative humidity and barometric pressure averaged over the relevant lag period.

Finally, we investigated the long-term associations of log-FeNO with PM_10_, NO_2,_ summer O_3_ (10 μg/m^3^ increment), PM_2.5_ (5 μg/m^3^ increment), and air temperature (1 °C) using separate multiple linear regression models adjusted for sex, age, BMI, smoking habits, allergic sensitization, and the hierarchical disease indicator.

All analyses were performed using STATA 18.0 (StataCorp, College Station, Texas).

## Results

### Study population

Of the 2,961 individuals invited to the clinical examination, 1,322 attended (response rate = 45%). Address geocoding was accurate for 1,308 of them, and 1,017 subjects had FeNO measurements (**Figure 1**).

Males accounted for 52.2% and 49.3% of individuals with and without chronic respiratory disease, respectively (**Table 1**). Individuals with respiratory diseases were younger (*P* < 0.001) and more likely to have allergic sensitization (78.7% vs 34.2%, *P* < 0.001). The two groups had a similar distribution of other characteristics, including education, smoking habits, and BMI.

### FeNO seasonality

Among the 605 individuals without chronic respiratory diseases, the median FeNO was 15.2 ppb (I–III quartile: 10.1–23.2). Participants with asthma (n = 152) had the highest median FeNO (21.1 ppb, 14.5–40.4), followed by participants with rhinitis (n = 197; 17.5 ppb, 9.8–35.7) and chronic bronchitis/COPD (n = 63; 12.6 ppb, 7.8–25.5).

Participation by calendar month is illustrated in **Supplementary Table 1**; https://links.lww.com/EE/A438. Visits were conducted both during the morning (8–11 am) and the afternoon (12–15 pm). The timing of the visit was reasonably balanced across seasons (afternoon visits: 28% in spring, 37% in summer, 35% in autumn, and 35% in winter) and between cases and controls (33% vs. 34%, respectively). The distribution of FeNO measurements did not differ by visit timing (**Supplementary Figure 2**; https://links.lww.com/EE/A438).

**Figure 2.**
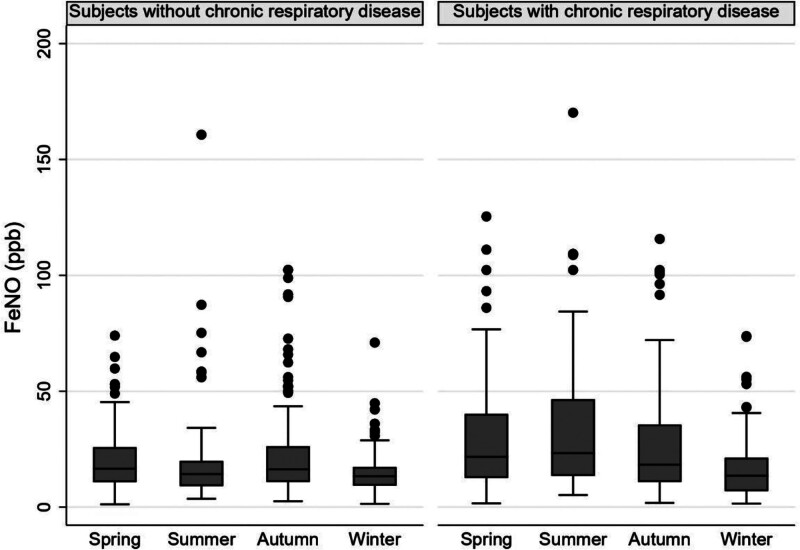
Raw distributions of FeNO concentrations for individuals without and with chronic respiratory diseases, by season. For graphical clarity, the data point for a participant with rhinitis having a FeNO of 640 ppb in autumn was excluded.

FeNO levels varied across seasons both for individuals with and without chronic respiratory diseases (**Figure 2**). Among participants with chronic respiratory diseases, after adjusting for individual characteristics, higher FeNO levels were observed during summer (median: 23.3 ppb, I–III quartile: 13.5–46.5) (*P* < 0.001) (**Supplementary Table 2;** https://links.lww.com/EE/A438). In contrast, among participants without respiratory diseases, FeNO levels were higher in spring (16.5 ppb, 10.8–25.8) and autumn (16.3 ppb, 10.9–26.2) (*P* = 0.003).

**Figure 3.**
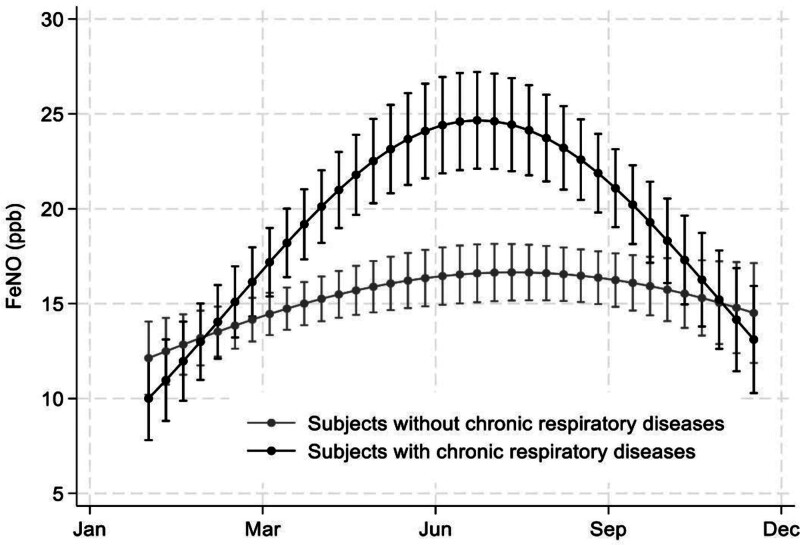
Adjusted seasonal variation of FeNO concentrations in individuals with and without chronic respiratory diseases. Marginal estimates from a linear regression model of log-FeNO including the binary disease indicator, sex, age, BMI, smoking, allergic sensitization, day, day × disease status (binary), day^2^, and day^2^ × disease status.

In the adjusted analysis, we observed higher FeNO levels during the warm season compared to the cold season, and this seasonal pattern differed between participants with and without chronic respiratory diseases (*P* for interaction = 0.001). In fact, it was more pronounced in individuals with respiratory diseases, but it was also observed to a lesser extent in those without (**Figure 3**). The analysis adjusted for pollen exposure and allergic sensitization provided consistent results (**Supplementary Figure 1;** https://links.lww.com/EE/A438).

### Short-term associations

The typical daily values of PM_10_ concentration, air temperature, barometric pressure, and relative humidity over the study period are shown in **Supplementary Figure 3;** https://links.lww.com/EE/A438.

**Supplementary Table 3**; https://links.lww.com/EE/A438 shows descriptive statistics for short-term exposure to PM_10_ and air temperature. Mean PM_10_ concentration was 38.5 μg/m^3^, and mean air temperature was 14 °C, regardless of the averaging period. There were moderate negative correlations between PM_10_ and temperature, with Spearman’s coefficients ranging between −0.48 (lag 0–6) and −0.34 (lag 0–1) (**Supplementary Figure 4**; https://links.lww.com/EE/A438).

In single-exposure models, higher PM_10_ concentrations were associated with higher FeNO levels at lag 0–1 (**Table 2**), although estimates were generally imprecise. The association became stronger when adjusting for air temperature: a 10 μg/m^3^ higher PM_10_ concentration was associated with a 3% higher FeNO at lag 0–1 (RGM: 1.03; 95% CI: 1.00, 1.06, *P* = 0.044). Considering a mean FeNO of 22 ppb in the study sample, this translates to roughly a 1.3 ppb higher FeNO per SD (20 μg/m^3^). The estimated associations were weaker and less precise at lag 0–3 and 0–6 (**Table 2**).

**Table 2.** Estimated associations between environmental exposures and FeNO concentrations for different exposure averaging windows^a^

	Lag 0–1	*P*	Lag 0–3	*P*	Lag 0–6	*P*
	RGM (95% CI)		RGM (95% CI)		RGM (95% CI)	
PM_10_ (per 10 μg/m^3^)	1.02 (1.00, 1.05)	0.091	1.01 (0.98, 1.04)	0.387	1.01 (0.98, 1.05)	0.354
	1.03 (1.00, 1.06)^b^	0.044	1.02 (0.99, 1.05)^b^	0.284	1.02 (0.99, 1.05)^b^	0.300
Temperature (per 10 °C)	0.91 (0.79, 1.05)	0.204	0.92 (0.79, 1.08)	0.328	0.92 (0.79, 1.09)	0.335
	0.88 (0.76, 1.02)^b^	0.100	0.91 (0.77, 1.07)^b^	0.244	0.92 (0.78, 1.08)^b^	0.285

a Ratios of geometric means (RGM) were obtained by exponentiating multiple linear regression coefficients for log-FeNO. All regression models were adjusted for disease status (hierarchical definition), sex, age, BMI, smoking, allergic sensitization, day, and day^2^.

b Mutually adjusted association: PM_10_ and temperature in the same model.

There was an indication of a negative association between air temperature and FeNO: after adjusting for seasonality, a 10 °C higher air temperature at lag 0–1 was associated with a 12% lower FeNO concentration, but with wide confidence intervals (RGM: 0.88; 95% CI: 0.76, 1.02, *P* = 0.100). This translates to roughly a 2.1 ppb lower FeNO per SD (8° C).

When considering the heterogeneity in seasonality between subjects with and without chronic respiratory diseases (i.e., including day × disease status and day^2^ × disease status), the associations with FeNO estimated at lag 0–1 for PM_10_ and temperature were consistent with the main analysis (**Supplementary Table 4**; https://links.lww.com/EE/A438).

Clinical examinations were primarily conducted between 2008 and 2010 (**Supplementary Table 5**; https://links.lww.com/EE/A438). Including adjustment for year (**Supplementary Table 6**; https://links.lww.com/EE/A438) did not alter the study findings.

Air temperature was moderately correlated with relative humidity (Spearman’s rho: −0.50) and poorly correlated with barometric pressure (0.20) (**Supplementary Figure 5**; https://links.lww.com/EE/A438). Adjusting for these additional variables provided consistent results (**Supplementary Tables 7**; https://links.lww.com/EE/A438). Relative humidity and barometric pressure were not associated with FeNO (**Supplementary Table 8**; https://links.lww.com/EE/A438).

### Long-term associations

Annual mean (±SD) exposures to PM_10_ and PM_2.5_ were 32.9 ± 4.0 μg/m^3^ and 24.5 ± 2.8 μg/m^3^, respectively (**Supplementary Table 9**; https://links.lww.com/EE/A438). There was considerable variability in NO_2_ levels. Indeed, annual exposure to NO_2_ ranged between 9.1 and 40.3 μg/m^3^, with a mean of 29.2 ± 6.8 μg/m^3^. Exposure to summer O_3_ ranged between 62.0 and 103.4 μg/m^3^, with a mean of 72.4 ± 5.4 μg/m^3^. Annual mean air temperature was 14.8 ± 0.6 °C.

We found no evidence of association between long-term exposure to air pollution, air temperature, and FeNO concentrations (**Table 3**).

**Table 3.** Estimated associations between long-term air pollutant exposures, air temperature, and FeNO concentrations

	Crude analysis^a^		Adjusted analysis^b^	
	RGM (95% CI)	*P*	RGM (95% CI)	*P*
PM_10_ (per 10 μg/m^3^)	1.01 (0.90, 1.13)	0.908	0.99 (0.88, 1.11)	0.877
PM_2.5_ (per 5 μg/m^3^)	1.00 (0.92, 1.09)	0.981	0.99 (0.91, 1.08)	0.841
NO_2_ (per 10 μg/m^3^)	1.00 (0.94, 1.08)	0.916	1.00 (0.93, 1.07)	0.971
Summer O_3_ (per 10 μg/m^3^)	0.95 (0.87, 1.03)	0.229	0.95 (0.87, 1.03)	0.225
Temperature (per 1 °C)	1.01 (0.94, 1.09)	0.737	1.00 (0.93, 1.08)	0.950

Ratios of geometric means (RGM) were obtained by exponentiating multiple linear regression coefficients for log-FeNO.

a Adjusted for disease status (hierarchical definition).

b Adjusted for disease status (hierarchical definition), sex, age, BMI, smoking, and allergic sensitization.

## Discussion

We observed significant seasonal variations in FeNO levels in population-based samples of individuals with and without chronic respiratory diseases. Higher FeNO concentrations were found during the warmer months compared to the colder months among individuals with asthma, chronic bronchitis/COPD, or rhinitis. To a lesser extent, a similar seasonal pattern was seen among individuals without these diseases, suggesting an underlying seasonal component in airway inflammation. After adjusting for seasonality, PM_10_ exposure was associated with FeNO concentrations, with a 3% increase per 10 μg/m^3^ increment at lag 0–1. No long-term associations were detected between air pollution exposure and FeNO concentrations.

### FeNO seasonality

A marked seasonal variation was observed among individuals with chronic respiratory diseases. However, a comparable, albeit attenuated, seasonal variation was also noted in participants without these conditions. This suggests that FeNO fluctuations may not be solely attributable to underlying diagnoses.

One reason for the seasonal pattern in FeNO could be the concomitant exposure to airborne allergenic pollen. This pattern persisted after adjusting for a composite indicator of pollen exposure among individuals sensitized to the relevant pollen types. This suggests that additional seasonal factors, beyond pollen alone, contribute to the observed temporal variability.

A study on children with asthma in the United States found that FeNO levels were highest in autumn, followed by spring, winter, and summer.^30^ FeNO levels have been shown to vary across the day and seasonally in healthy adults as well.^31^ These findings align with our study, suggesting a seasonal variation in bronchial inflammation that may differ across geographical areas and populations.

### Short-term associations

We accounted for FeNO seasonality in analyzing short-term associations with PM_10_ concentrations and air temperature. This initial step aimed to eliminate confounding from intra-annual long-term variability, including the typical variation of air pollutants across the months of the year (e.g., higher traffic-related pollutants during winter and higher O_3_ levels in summer).^32^ This approach allows estimation of the day-to-day effects of PM_10_ within seasons.

Based on the mechanism of NO formation in the respiratory epithelium,^13,33^ FeNO levels exhibit pronounced sensitivity to oxidizing agents and allergens. Air pollution triggers inflammatory responses and impairs immune function, whereas temperature primarily intensifies the impact of disease vectors.^10^

We found that PM_10_ exposure in the days immediately preceding measurement was associated with higher FeNO levels. In contrast, air temperature showed an inverse pattern: after accounting for the underlying seasonality in FeNO, higher temperatures at lag 0–1 were related to lower FeNO, although the confidence intervals were wide (RGM: 0.88, 95% CI: 0.76, 1.02, per 10 °C). This suggests that, while temperature can influence airway physiology in the short term, its association with FeNO appears less consistent and more uncertain than that of PM_10_. Indeed, the estimated absolute change was small (in the order of a few ppb) and therefore below the typical inter-day biological variation in FeNO among healthy adults (usually around 20%).^34^ However, even modest shifts in individual FeNO can translate into meaningful impacts at the population level when exposure is widespread and affects large groups simultaneously.

Our findings are consistent with some, but not all, previous studies, which have primarily examined vulnerable age groups, including children and adolescents^35–37^ and the elderly.^15,38,39^ Most studies on the relationship between air pollution and FeNO have been conducted in countries with high levels of air pollution in the Eastern Hemisphere.^40^ This may partly explain why the estimated associations were higher in these studies compared to ours. For example, Chen et al^41^ reported an average increase in FeNO of 5.6 ppb (95% CI: 1.5, 11.0 ppb) per IQR increment of 34.8 µg/m^3^ in PM_10_ exposure in elderly individuals, corresponding to a 1.6 ppb increase per 10 µg/m^3^. Since the average FeNO concentration among participants was 28.5 ppb, this represents a 5.6% increase in FeNO per 10 µg/m^3^. The stronger association observed in Chen et al may be related to higher daily PM_10_ concentrations (53 µg/m^3^) than in our study (38.5 µg/m^3^). Areal et al^42^ found that higher PM_2.5_, O_3_, and NO_2_ concentrations were associated with increased FeNO in adolescents. However, in contrast with our study, they found a positive association with air temperature and a negative association with relative humidity. Zhao et al^43^ reported a 13% increase in FeNO per IQR increase in NO_2_ exposure on the measurement day, corresponding to a 1.6 ppb increase per 10 μg/m^3^. In contrast, some research has shown no significant relationships.^15,43–45^ A systematic review and meta-analysis concluded that short-term exposure to air pollution is associated with increased FeNO, supporting the hypothesis that airway inflammation mediates the respiratory effects of pollution.^46^

### Long-term associations

Our results revealed no association between long-term exposure to air pollutants and FeNO levels, suggesting that FeNO may be more sensitive to short-term than long-term exposure to air pollution. It is possible that FeNO levels return to baseline after a temporary exposure peak, with transient inflammation not persisting over time. We cannot exclude that the lack of association may be due to exposure misclassification, given the mismatch between the periods of long-term exposure assignment (2013–2015) and FeNO measurement (2007–2010). Nonetheless, annual PM_10_ exposures estimated for different periods were very highly correlated, as illustrated in **Supplementary Figure 6**; https://links.lww.com/EE/A438, consistent with previous findings.^18^ Furthermore, contrasts in air pollution exposures have been shown to remain stable over long periods.^47,48^

### Limitations

One study limitation is the uneven distribution of visits throughout the year, which may have compromised the seasonal representativeness of the data. However, efforts were made to mitigate this potential bias by inviting both individuals with and without respiratory diseases in parallel. Moreover, including the year as an additional adjustment variable did not affect our conclusions. Another potential limitation is self-selection bias, as individuals with respiratory diseases may have declined participation or rescheduled their visits based on their health status, potentially influencing the sample composition. The small sample size of the disease groups also limited the study’s statistical power. Furthermore, exposure assessment was based solely on outdoor residential air pollution concentrations and air temperature, without accounting for indoor exposures or personal activity patterns.

## Conclusion

Short-term exposure to PM_10_ and air temperature can influence FeNO levels regardless of season, whereas seasonality may affect bronchial inflammation even in healthy individuals. These findings support FeNO as a sensitive biomarker of environmental respiratory effects, emphasizing the need to consider seasonal and pollution-related variability in both research and clinical practice. Manufacturers of FeNO measurement devices should consider short-term fluctuations in particulate matter and ambient temperature when designing instruments and developing measurement algorithms. Overlooking seasonality and environmental exposures may affect clinical decision-making. Therefore, environmental variability should be accounted for to enhance the reliability and accuracy of long-term FeNO monitoring.

## Conflicts of interest statement

The authors declare that they have no conflicts of interest with regard to the content of this report.

## ACKNOWLEDGMENTS

*We express our gratitude to Dr Francesco Domenichini, a meteorologist from the regional environmental protection agency (ARPAV), for kindly providing monitoring data on relative humidity and barometric pressure.*

*The members of BIGEPI Group are Anna Antonietta Angino, Sandra Baldacci, Sara Maio, Giuseppe Sarno, Ilaria Stanisci, Sofia Tagliaferro, Giovanni Viegi (Institute of Clinical Physiology, CNR, Pisa); Salvatore Fasola, Stefania La Grutta (Institute of Translational Pharmacology, CNR, Palermo); Carla Ancona, Lisa Bauleo, Giulia Cesaroni, Paola Michelozzi, Matteo Renzi, Massimo Stafoggia (Department of Epidemiology SSR Lazio/ASL Rome 1); Giuseppe Costa, Nicolás Zengarini (Regional Public Health Observatory, ASL TO3, Collegno, Turin); Simone Giannini, Andrea Ranzi (Regional Agency for Prevention, Environment and Energy of Emilia-Romagna); Letizia Bartolini, Paolo Giorgi Rossi, Marta Ottone (Reggio Emilia AUSL-IRCCS); Nicola Caranci, Chiara Di Girolamo (Regional Health and Social Agency of Emilia-Romagna); Lucia Bisceglia (Strategic Regional Agency for Health and Social of Puglia); Achille Cernigliaro, Salvatore Scondotto (Department of Health and Epidemiological Observatory, Regional Health Authority of Sicily Region); Francesca Locatelli, Pierpaolo Marchetti, Alessandro Marcon, Lorena Torroni, Giuseppe Verlato (Department of Diagnostics and Public Health, University of Verona); Claudio Gariazzo, Alessandro Marinaccio, Stefania Massari (INAIL, Department of Occupational and Environmental Medicine, Roma); Camillo Silibello, Gianni Tinarelli (ARIANET S.r.l.).*

## Supplementary Material


