# Supplementary material for: Seasonality and environmental determinants of exhaled nitric oxide in individuals with and without chronic respiratory diseases
Source: Environ Epidemiol. 2026 Jun 30;10(4):e501. doi: 10.1097/EE9.0000000000000501 (PMC13318099; doi:10.1097/EE9.0000000000000501)
Supplement: Supplementary file 1 [file ee9-10-e501-s001.pdf]

SUPPLEMENTARY MATERIALS

Seasonality and environmental determinants of exhaled nitric oxide in individuals with and without chronic respiratory diseases

Lorena Torroni, Francesca Locatelli, Pierpaolo Marchetti, Sandra Baldacci, Claudio Gariazzo, Sara Maio, Camillo Silibello, Gianluca Spiteri, Massimo Stafoggia, Giovanni Viegi, Giuseppe Verlato, Alessandro Marcon, on behalf of the BIGEPI group.

Supplementary methods

Allergic sensitisation

Allergic sensitisation was defined as the presence of at least one positive skin prick test among inhaled allergens (*Cupressus arizonica*, Graminacee mix, *Artemisia vulgaris*, *Ambrosia artemisifolia*, *Alternaria tenuis*, *Parietaria Judaica*, *Corylus avellana*, *Olea europea*, *Betula verrucosa*, *Cladosporium herbarum*, Dog dander, Cat hair, *Dermatophagoides pteronyssinus*, *Dermatophagoides farinae*) (ALK diagnostics, Denmark). A test was considered positive if the mean diameter of the wheal was 3 mm greater than the negative control after 20 minutes.

Measurement of Fractional exhaled Nitric Oxide

FeNO measurements were performed at a flow rate of 50 mL/s, expressed in parts per billion (ppb), before spirometry, in accordance with international guidelines, using a chemiluminescence analyser (CLD88; Ecomedics Switzerland). Subjects were instructed to take a deep breath, hold it briefly, then slowly exhale into a mouthpiece fitted with a 0.2-µm bacterial filter. This filter was part of an exhalation circuit that included an ultrasonic flow meter, a one-way valve, and a sampling port. NO was directly sampled into the analyser at 250 mL/min through a Teflon side arm tube connected to the sampling port. The tube was 60 cm long with an internal diameter of 1/8 inch. Both expiratory flow and FeNO values were displayed simultaneously on a connected computer. FeNO is measured based on the photochemical reaction between NO and ozone (O_3). The device generates O_3, which reacts with NO to produce energised NO_2, emitting photons (light) when returning to its normal energy state. The emitted light correlates with the concentration of NO in the exhaled air. During each test, the NO concentration in the inhaled ambient air was monitored, and measurements were discarded if ambient levels exceeded 30 ppb. The influence of ambient NO was further minimised by placing an NO-scrubbing filter into the inspiratory limb of the collection apparatus.

Exposure models

Time series of daily concentrations of the primary air pollutants (PM_{10}, $PM_{2.5}$, NO_2, and O_3) and air temperature were associated with the geocoded individual addresses. These exposure time series were obtained using validated models previously developed in the BEEP (Big data in Environmental and occupational Epidemiology) and BIGEPI (Use of big data to assess the acute and chronic health effects of air pollution in the Italian population; https://bigepi.it/) projects.[1] In brief, daily PM_{10} (years 2006–2015) and $PM_{2.5}$ (years 2013–2015) concentrations were obtained for each 1 x 1 km cell in the study domain using spatiotemporal models based on machine learning Random Forest algorithms (MLRF).[2,3] These models used spatial and spatiotemporal variables, including aerosol optical depth (AOD), land use, meteorological data, emission data, and resident population.

NO_2 and O_3 concentrations were derived using an integrated approach that combined a chemical transport model with machine-learning techniques.[4] As described elsewhere, simulations were performed at a 5 km spatial resolution using the Flexible Air Quality Regional Model (FARM). Results from these simulations, along with other spatial and spatiotemporal data, such as population, land use, surface greenness, and road networks, were used as predictors by an MLRF algorithm to produce daily concentrations at a 1-km resolution.[4,5]

Supplementary Table 1. Number of participants by disease status and month of the clinical examination.

	**N. without chronic respiratory diseases**	**N. with chronic respiratory diseases**	*N. with asthma*	*N. with chronic bronchitis/ COPD*	*N. with rhinitis*
**January**	54	24	*9*	*3*	*12*
**February**	69	33	*12*	*6*	*15*
**March**	58	23	*12*	*3*	*8*
**April**	70	42	*9*	*9*	*24*
**May**	58	48	*20*	*4*	*24*
**June**	54	45	*13*	*9*	*23*
**July**	48	38	*13*	*3*	*22*
**August**	66	59	*27*	*11*	*21*
**September**	46	43	*21*	*6*	*16*
**October**	48	32	*8*	*7*	*17*
**November**	34	25	*8*	*2*	*15*
**Total**	**605**	**412**	*152*	*63*	*197*

Supplementary Table 2. Seasonal distribution of FeNO concentrations (ppb) by disease status.

	**n**	**Subjects without chronic respiratory diseases** Median (I-III quartile)	**Subjects with chronic respiratory diseases** [a] Median (I-III quartile)
Spring	299	16.5 (10.8-25.8)	21.7 (12.6-40.2)
Summer	185	14.2 (9.1-19.9)	23.3 (13.5-46.5)
Autumn	294	16.3 (10.9-26.2)	18.5 (10.9-35.7)
Winter	239	13.3 (9.3-17.3)	13.6 (6.9-21.3)
p-value[b] , unadjusted		<0.001	<0.001
p-value[b] , adjusted[c]		0.003	<0.001

[a] Asthma and/or chronic bronchitis/COPD and/or rhinitis

[b] p-values from likelihood ratio tests comparing linear regression models of log-FeNO with and without season

[c] analysis adjusted for sex, age, BMI, smoking, and allergic sensitisation

Supplementary Table 3. Distribution of environmental exposure variables averaged over different exposure windows (n=1017).

Exposure	Averaging period	Min, Max	Mean ± SD
PM_{10} (µg/m^3)	*Lag 0-1*	8.1, 125.8	38.5 ± 20.6
	Lag 0-3	8.5, 123.7	38.2 ± 19.9
	Lag 0-6	9.3, 114.6	38.5 ± 18.6
Temperature (°C)	*Lag 0-1*	-4.8, 30.0	14.1 ± 7.8
	Lag 0-3	-3.6, 29.3	14.0 ± 7.7
	Lag 0-6	-2.6, 28.9	14.0± 7.7
Relative humidity (%)	*Lag 0-1*	28.5, 100.0	76.3±13.0
	Lag 0-3	38.0, 100.0	76.3±11.8
	Lag 0-6	43.4, 99.8	76.5±10.5
Barometric pressure (mbar)	*Lag 0-1*	965.7, 1014.5	995.2±6.8
	Lag 0-3	974.9, 1011.6	995.2±6.1
	Lag 0-6	978.8, 1009.9	995.2±5.4

Supplementary Table 4. Estimated associations between environmental exposures and FeNO concentrations for different exposure averaging windows: sensitivity analysis considering the heterogeneity in FeNO seasonal patterns across subjects with and without respiratory diseases (n=974)[a].

	**Lag 0-1**		**Lag 0-3**		**Lag 0-6**	
	**RGM (95% CI)**	**p**	**RGM (95% CI)**	**p**	**RGM (95% CI)**	**p**
**PM_{10}** (per 10 µg/m^3)						
Model 1	1.02 (1.00-1.05)	0.109	1.01 (0.99-1.04)	0.324	1.02 (0.99-1.05)	0.287
Model 2	1.03 (1.00-1.05)	0.056	1.02 (0.99-1.05)	0.232	1.02 (0.99-1.05)	0.239
**Temperature** (per 10 °C)						
Model 1	0.91 (0.79-1.06)	0.234	0.92 (0.79-1.08)	0.325	0.92 (0.78-1.08)	0.322
Model 2	0.89 (0.76-1.03)	0.113	0.91 (0.77-1.06)	0.232	0.91 (0.78-1.07)	0.266

[a] Ratios of Geometric Means (RGM) were obtained by exponentiating multiple linear regression coefficients of log-FeNO. Model 1: Regression models adjusted for sex, age, BMI, smoking, allergic sensitisation, day, disease status (binary), day × disease status (binary), day^2, and day^2 × disease status (binary); Model 2: Model 1 with PM_{10} and temperature included in the same model.

Supplementary Table 5. Distribution of medical examinations by study year.

	N	%
**2008**	238	23.40
**2009**	432	42.48
**2010**	289	28.42
**2011**	26	2.56
**2013**	5	0.49
**2014**	27	2.65
**Total**	1017	100.00

Supplementary Table 6. Estimated associations between environmental exposures and FeNO concentrations for different exposure averaging windows: sensitivity analysis including participation year as an additional adjustment variable[a].

	Lag 0-1 (n=974)		Lag 0-3 (n=974)		Lag 0-6 (n=974)	
	**RGM (95% CI)**	**p**	**RGM (95% CI)**	**p**	**RGM (95% CI)**	**p**
**PM_{10}** (per 10 µg/m^3)						
Model 1	1.02 (0.99-1.05)	0.123	1.01 (0.98-1.04)	0.464	1.01 (0.98-1.05)	0.388
Model 2	1.03 (1.00-1.05)	0.057	1.01 (0.99-1.04)	0.317	1.02 (0.99-1.05)	0.309
**Temperature** (per 10 °C)						
Model 1	0.90 (0.77-1.04)	0.153	0.90 (0.77-1.05)	0.192	0.90 (0.76-1.06)	0.193
Model 2	0.87 (0.75-1.01)	0.070	0.89 (0.76-1.04)	0.141	0.89 (0.76-1.05)	0.159

[a] Ratios of Geometric Means (RGM) were obtained by exponentiating multiple linear regression coefficients of log-FeNO. CI: Confidence interval. Model 1: Regression models adjusted for disease status (hierarchical definition), sex, age, BMI, smoking, allergic sensitisation, day, day^2, and year of participation (categorical); Model 2: Model 1 with PM_{10} and temperature included in the same model.

Supplementary Table 7. Estimated associations between environmental exposures and FeNO concentrations for different exposure averaging windows: sensitivity analysis including relative humidity and barometric pressure as additional adjustment variables[a].

	Lag 0-1		Lag 0-3		Lag 0-6	
	(n=945)		(n=952)		(n=952)	
	**RGM (95% CI)**	**p**	**RGM (95% CI)**	**p**	**RGM (95% CI)**	**p**
**PM_{10}** (per 10 µg/m^3)						
Model 1	1.02 (0.99-1.06)	0.116	1.01 (0.98-1.05)	0.440	1.01 (0.98-1.05)	0.453
Model 2	1.03 (1.00-1.05)	0.075	1.02 (0.98-1.05)	0.360	1.02 (0.98-1.05)	0.411
**Temperature** (per 10 °C)						
Model 1	0.93 (0.80-1.08)	0.318	0.93 (0.80-1.09)	0.389	0.93 (0.79-1.10)	0.397
Model 2	0.90 (0.77-1.05)	0.194	0.92 (0.78-1.08)	0.321	0.93 (0.79-1.09)	0.362

[a] Ratios of Geometric Means (RGM) were obtained by exponentiating multiple linear regression coefficients of log-FeNO. CI: Confidence interval. Model 1: Regression models adjusted for disease status (hierarchical definition), sex, age, BMI, smoking, allergic sensitisation, day, day^2, relative humidity, and barometric pressure; Model 2: Model 1 with PM_{10} and temperature included in the same model.

Supplementary Table 8. Estimated associations between relative humidity and barometric pressure, measured at the closest monitor and averaged over different exposure windows, and FeNO concentrations[a].

	Lag 0-1		Lag 0-3		Lag 0-6	
	(n=945)		(n=952)		(n=952)	
	**RGM (95% CI)**	**p**	**RGM (95% CI)**	**p**	**RGM (95% CI)**	**p**
Relative humidity (per 10%)	0.98 (0.94-1.03)	0.482	0.98 (0.93-1.03)	0.375	0.97 (0.92-1.04)	0.549
Barometric pressure (per 5 mbar)	1.01 (0.97-1.06)	0.520	1.01 (0.97-1.06)	0.547	1.02 (0.96-1.07)	0.398

[a] Ratios of Geometric Means (RGM) were obtained by exponentiating multiple linear regression coefficients for log-FeNO. CI: Confidence interval. All regression models were adjusted for disease status (hierarchical definition), sex, age, BMI, smoking, allergic sensitisation, day, day^2, daily PM_{10} exposure, and daily air temperature

Supplementary Table 9. Distribution of annual air pollution exposures (µg/m[3]) estimated for 2013-2015 (n=1017).

Exposure	Min-Max	Mean (± SD)
PM_{10}	11.2 - 35.8	32.9 ± 4.0
$PM_{2.5}$	9.1 - 26.7	24.5 ± 2.8
NO_2	9.1 - 40.3	29.2 ± 6.8
Summer O_3	62.0 - 103.4	72.4 ± 5.4
Air temperature	0.5 - 15.5	14.8 ± 0.6

Supplementary Figure 1. Estimated seasonal patterns of FeNO concentrations for individuals with and without chronic respiratory diseases: sensitivity analysis considering concomitant pollen exposures and individual allergic sensitisation status[a].

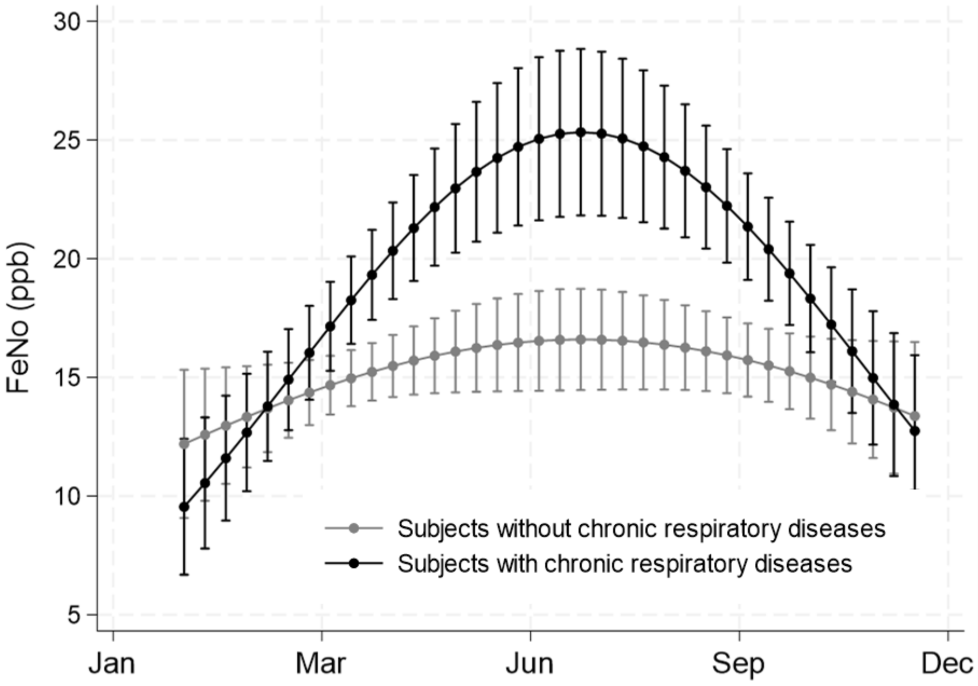

[a] Marginal estimates from a linear regression model of log-FeNO, including disease status (binary), sex, age, BMI, smoking, day, day × disease status (binary), day^2, day^2 × disease status (binary), and a combined indicator of pollen exposure and allergic sensitisation. This indicator classified the participants into four categories, as previously described:[6] I) unexposed and unsensitized; II) exposed or sensitised only (but not both); III) exposed and sensitised to one pollen species (e.g., individuals with grass monosensitisation having FeNO measured during the grass pollen season); and IV) exposed and sensitised to multiple pollen species.

Supplementary Figure 2. Distribution of FeNO measurements by visit timing [a].

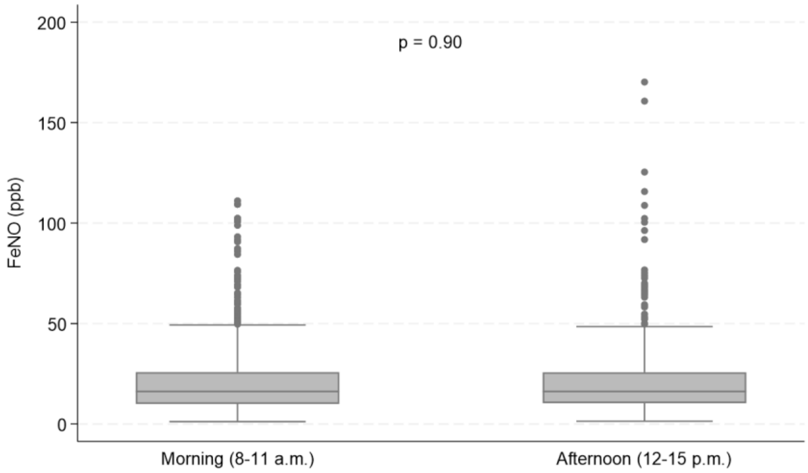

[a] For graphical clarity, one observation from an afternoon visit (approximately 600 ppb) was excluded; p-value from a Mann–Whitney rank-sum test.

Supplementary Figure 3. Average daily values of air temperature, PM_{10} concentration, barometric pressure, and relative humidity (panels A–D, respectively), calculated over the period 2008-2010.*

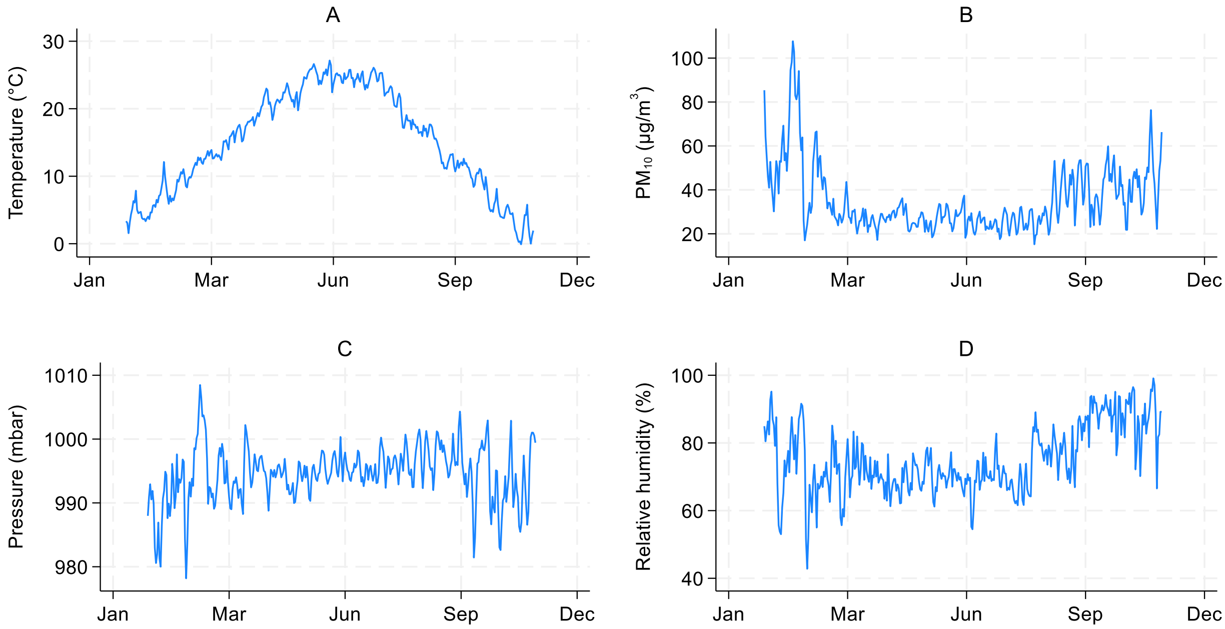

* This period was selected because most participants (96.9%) attended clinical examinations during these years.

Supplementary Figure 4. Scatterplots and Spearman's coefficients for the correlation between PM_{10} concentrations and air temperature averaged over different exposure windows.

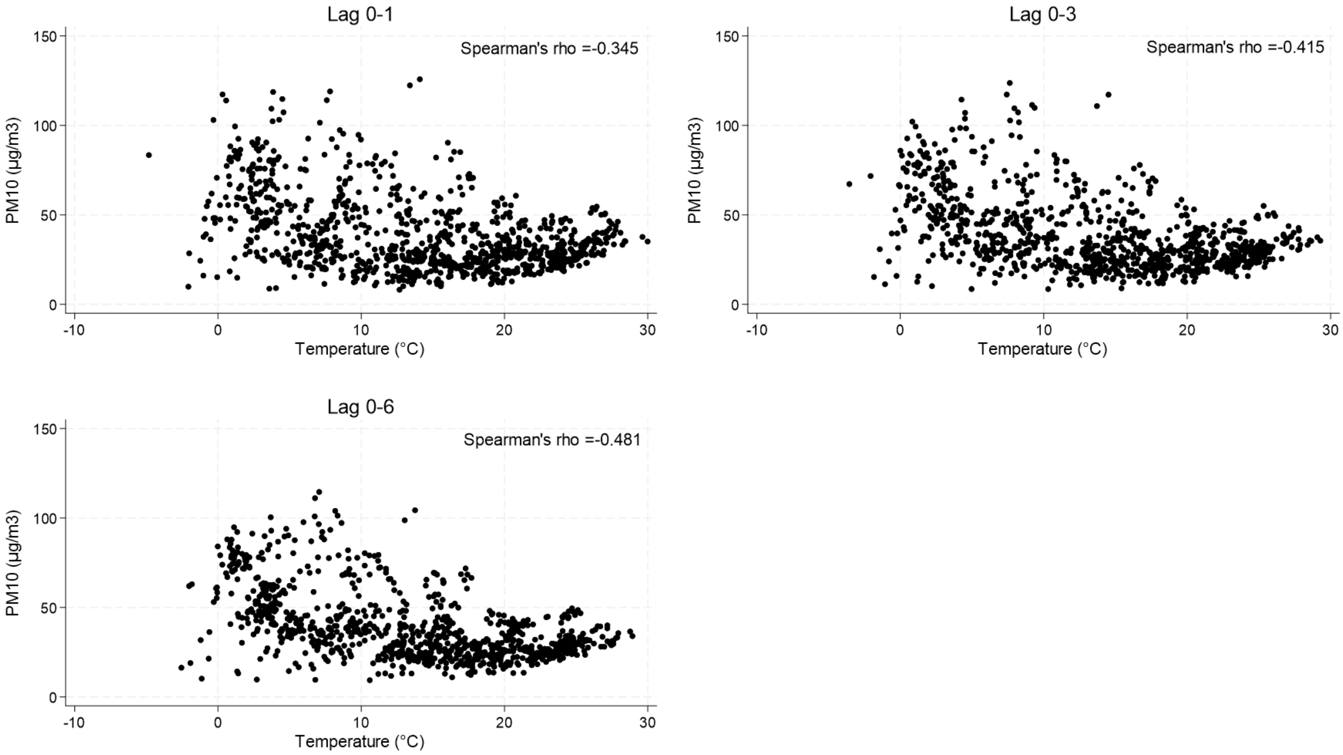

Supplementary Figure 5. Scatterplots and Spearman's coefficients for the correlation between relative humidity and air temperature (panel A), and between barometric pressure and air temperature (panel B) (lag 0-1).

A

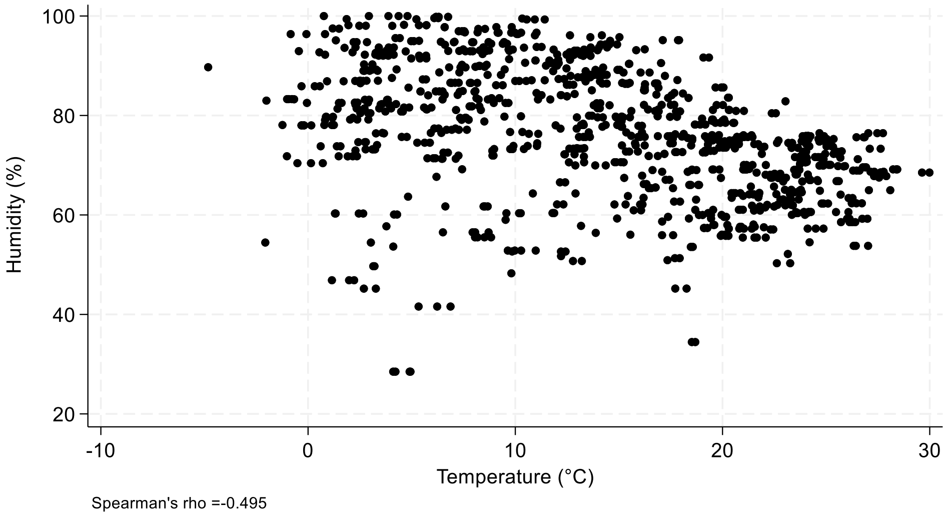

B

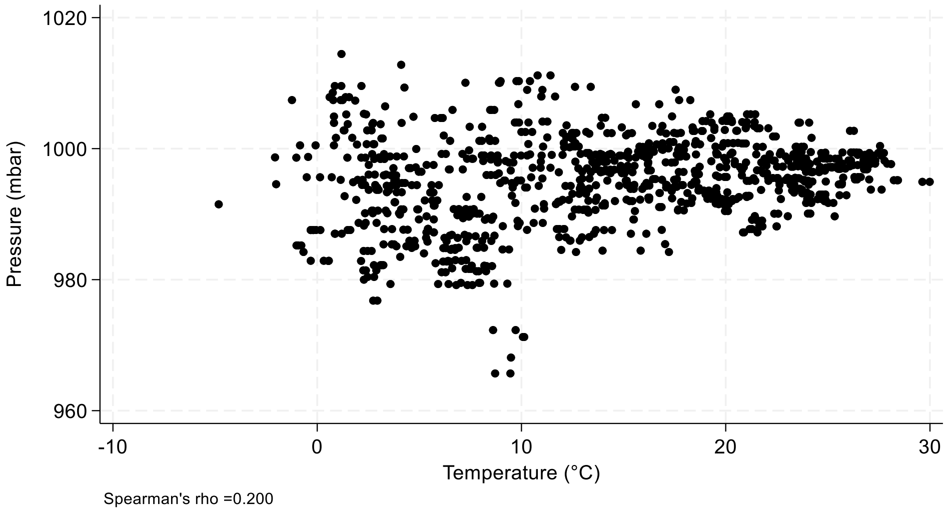

Supplementary Figure 6. Scatter plots and Pearson's coefficients for the pairwise correlations of PM_{10} concentrations and air temperature over different exposure averaging windows (n=1017).[a]

A

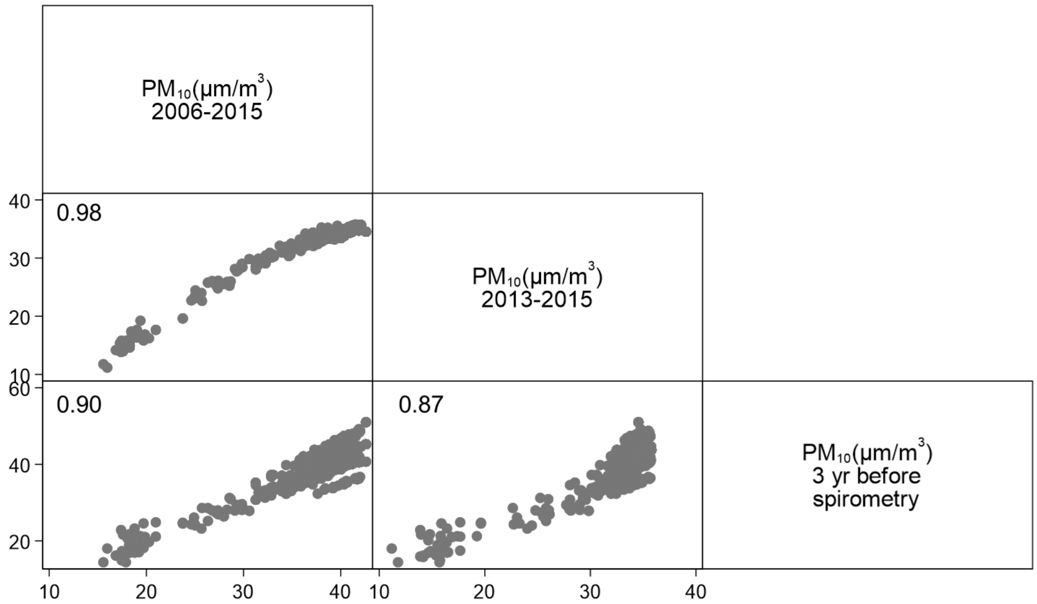

B

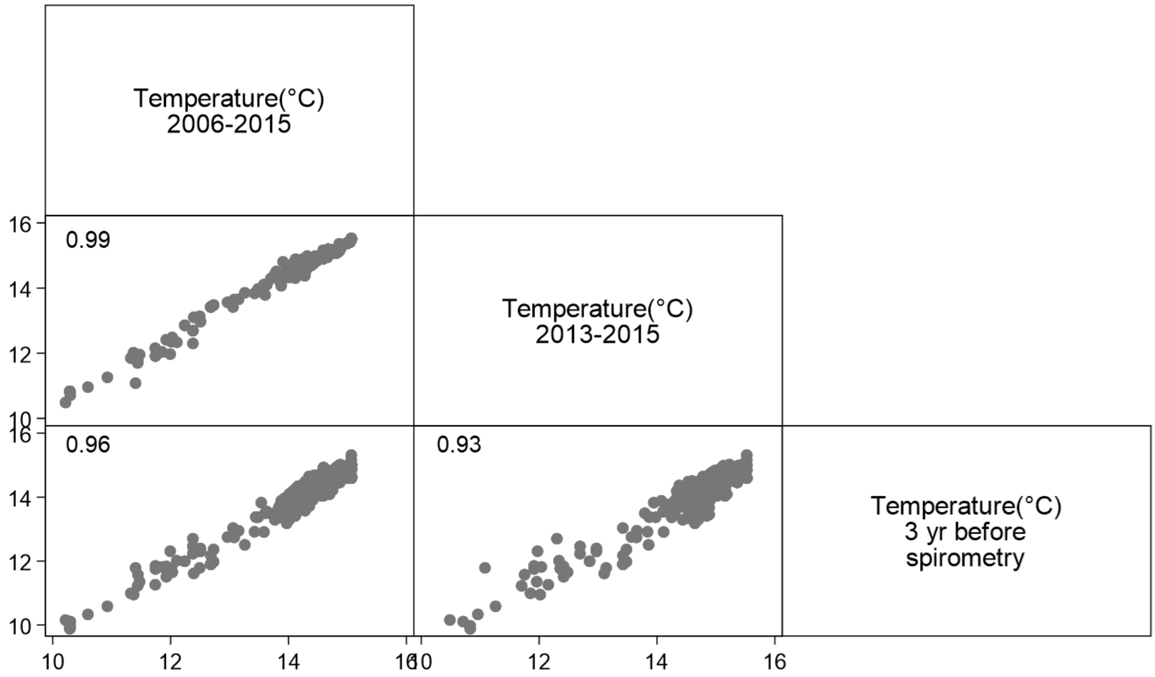

[a] 2006-2015 (all data available); 2013-2015 (common period available for all exposures); 3-year period before interview (e.g., 2008-2010 for a subject interviewed in 2010)

References

1 Maio S, Gariazzo C, Stafoggia M, Ancona C, Bisceglia L, Caranci N *et al.* BIGEPI project: environmental and health data. *Epidemiol Prev* 2023; **47**: 8–18.

2 Stafoggia M, Schwartz J, Badaloni C, Bellander T, Alessandrini E, Cattani G *et al.* Estimation of daily PM10 concentrations in Italy (2006–2012) using finely resolved satellite data, land use variables and meteorology. *Environ Int* 2017; **99**: 234–244.

3 Stafoggia M, Bellander T, Bucci S, Davoli M, de Hoogh K, de' Donato F *et al.* Estimation of daily PM10 and PM2.5 concentrations in Italy, 2013–2015, using a spatiotemporal land-use random-forest model. *Environ Int* 2019; **124**: 170–179.

4 Silibello C, Carlino G, Stafoggia M, Gariazzo C, Finardi S, Pepe N *et al.* Spatial-temporal prediction of ambient nitrogen dioxide and ozone levels over Italy using a Random Forest model for population exposure assessment Content courtesy of Springer Nature, terms of use apply. Rights reserved. *Air Qual Atmos Health* 2021; **14**: 817–829.

5 Marchetti P, Miotti J, Locatelli F, Antonicelli L, Baldacci S, Battaglia S *et al.* Long-term residential exposure to air pollution and risk of chronic respiratory diseases in Italy: The BIGEPI study. *Science of the Total Environment* 2023; **884**. doi:10.1016/j.scitotenv.2023.163802.

6 Olivieri M, Marchetti P, Murgia N, Nicolis M, Torroni L, Spiteri G *et al.* Natural pollen exposure increases in a dose-dependent way Fraction of exhaled Nitric Oxide (FeNO) levels in patients sensitized to one or more pollen species. *Clin Transl Allergy* 2022; **12**. doi:10.1002/clt2.12096.
